# Cone-wedge anchored surgical templates for stackable metal guide: a novel technique

**DOI:** 10.1186/s40729-024-00539-w

**Published:** 2024-05-31

**Authors:** Xueying Bai, Tao Wu, Yuxi Zhu, Chengyu Yang, Tiange Cheng, Yi Liu, Yi Zhou

**Affiliations:** 1 State Key Laboratory of Oral & Maxillofacial Reconstruction and Regeneration, Key Laboratory of Oral Biomedicine Ministry of Education, Hubei Key Laboratory of Stomatology, School & Hospital of Stomatology, Wuhan University, Wuhan, PR China; 2 Center for Prosthodontics and Implant Dentistry, Optics Valley Branch, School and Hospital of Stomatology; State Key Laboratory of Oral & Maxillofacial Reconstruction and Regeneration, Key Laboratory of Oral Biomedicine Ministry of Education, Hubei Key Laboratory of Stomatology, School & Hospital of Stomatology , Wuhan University, Wuhan University, Wuhan, PR China; 3 Department of Stomatology, Edong Healthcare Group, Huangshi Central Hospital, Affiliated Hospital of Hubei Polytechnic University, Huangshi, PR China; 4 Center for Prosthodontics and Implant Dentistry, Optics Valley Branch, School and Hospital of Stomatology; State Key Laboratory of Oral & Maxillofacial Reconstruction and Regeneration, Key Laboratory of Oral Biomedicine Ministry of Education, Hubei Key Laboratory of Stomatology, School & Hospital of Stomatology , Wuhan University, Wuhan, 430000 PR China

**Keywords:** Edentulous, Implant, Static computer-assisted implant surgery, Surgical guide, Templates

## Abstract

**Objective:**

To address the instability in implant surgical guides, this technique proposes an alternative anchoring mechanism in the stackable metal surgical guides utilizing cone-wedge anchors for improved stability.

**Methods:**

Postoperative implant position superimposed onto the preoperatively planned design using Mimics Medical 21.0 and Materialise Magics 24.0 to assess 3D coronal implant deviation, 3D apical implant deviation, and implant angular deviation.

**Results:**

Postoperative cone-beam computed tomography (CBCT) revealed a high level of precision in the implant placement, with an average 0.97 mm deviation at implant coronal region, 1.56 mm at implant apexes, and 2.95° angular deviation.

**Conclusion:**

This technique introduces a novel cone-wedge anchoring mechanism to enhance the stability of stackable metal surgical guide templates, addressing inherent instability issues. The utilization of this approach significantly improves the accuracy of implant placement procedures.

**Supplementary Information:**

The online version contains supplementary material available at 10.1186/s40729-024-00539-w.

## Introduction

Computer-assisted implant surgery (CAIS) is considered optimal for placing dental implants in the desired prosthetic and anatomical position [1, 2]. Owing to cone-beam computed tomography (CBCT) scanning [3], virtual planning software [4], computer-aided manufacturing technology (CAM) [5], and other digital technology development, virtual implant plans can be realized in clinical settings. Static surgical guides are vital mediums for transferring the preoperative virtually designed implant position into the patient’s mouth. Although surgical guides can significantly increase the precision of implant surgery, there is still an inevitable deviation between the preoperative design and postoperative position. The errors mainly originate from imaging collection [4], data transfers [6], template manufacturing [7], surgical template positioning [8], operator’s experience [9], drill macro-design [10], and drilling strategy (sleeve height, drilling distance, drilling key length) [11].

Surgical template fixation and stabilization during the implant bed preparation may have caused the high deviation in the final implant position [12]. Sequential implant surgical templates are typically utilized for full-arch edentulous patients, which are made up of several subset templates to function in scenarios such as template retention, bone reduction, or implant placement. These subset templates share the same retainer pins anchored on the alveolar bone. However, once the bone retainer pins are removed, the repositioning of the pins may be difficult. Therefore, a stackable surgical template was proposed [13]. Typically, a stackable surgical template possesses two parts: the base template and the super-structure templates. The base template is connected to the alveolar bone by retainer pins, and the super-structural templates need to connect to the base template. According to its function in surgery, the super-structural templates can be categorized into several subset templates such as positioning template, bone reduction template, and implant template. A stackable surgical template takes the strategy that the base template is precisely fixed on the alveolar bone by bone retainer pins with the help of the positioning template, and the upper-structure templates (such as the positioning template, the bone reduction template, and implant placement template) are anchored to the base template via different fixation approaches, correspondingly. For improved accuracy, some studies have focused on the fixation modification between the base template and the upper-structure templates, such as bead-anchored structure [8], magnetically retained structure [14–16], strut-based lattice structure [13], ball-snap structure [17], and pin-anchored structure [18].

This study proposes a novel cone-wedge anchorage technique to connect and stabilize the upper-structure templates to the base template and to improve surgical template fixation and stabilization.

## Technique

1. Extract the remaining teeth and fabricate a diagnostic denture after 3 months when the hard and soft tissue recovered (Fig. 1A, B). Attach the smile line and fiducial to the dentures which served as radiographic templates for CBCT scanning (Newtom VGi) using the dual scan technique (Fig. 1C, D, E) [19, 20].
  **Fig. 1:**
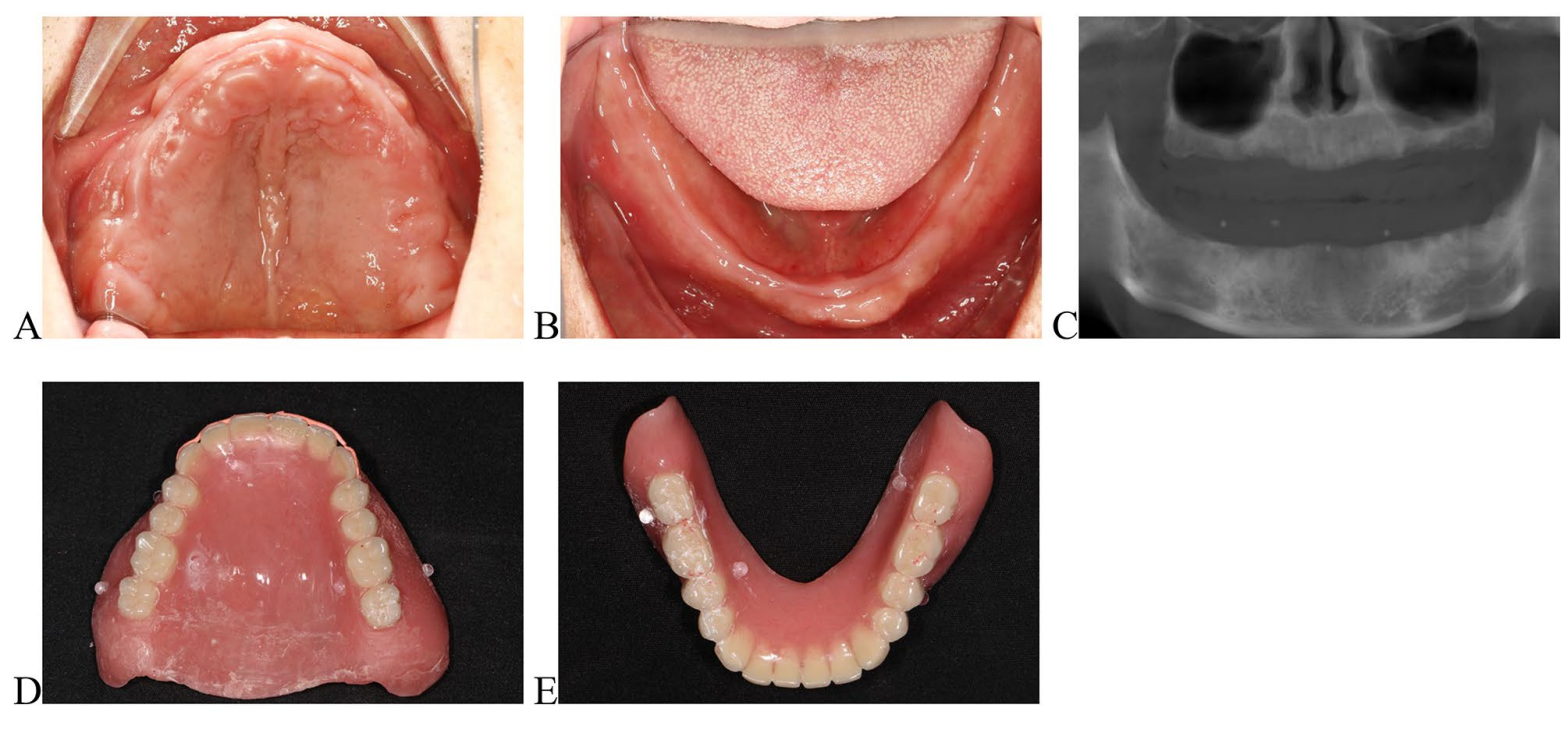
**A**, Preoperative intraoral photograph of maxillary. **B**, Preoperative intraoral photograph of mandibular. **C**, Radiographic examination. **D**, Maxillary diagnosis denture with smile line. **E**, Mandibular diagnosis denture
2. Import the Digital Imaging and Communications (DICOM) files into a computer-aided design (CAD) surgical implant planning program (3Shape 2020), and perform prosthetically driven computer-aided implant planning. Plan four dental implants with four fixation pins in the maxillary and two implants with three fixation pins in the mandible (Template Fixation Pin 1.5 × 32 mm, Inclusive). Import the surgical implant guide information using standard tessellation language (STL) files into the forward engineering software (Materialise 3-matic 20.0) and design the metallic retainer template and implant placement templates (Fig. 2A-D).
  **Fig. 2:**
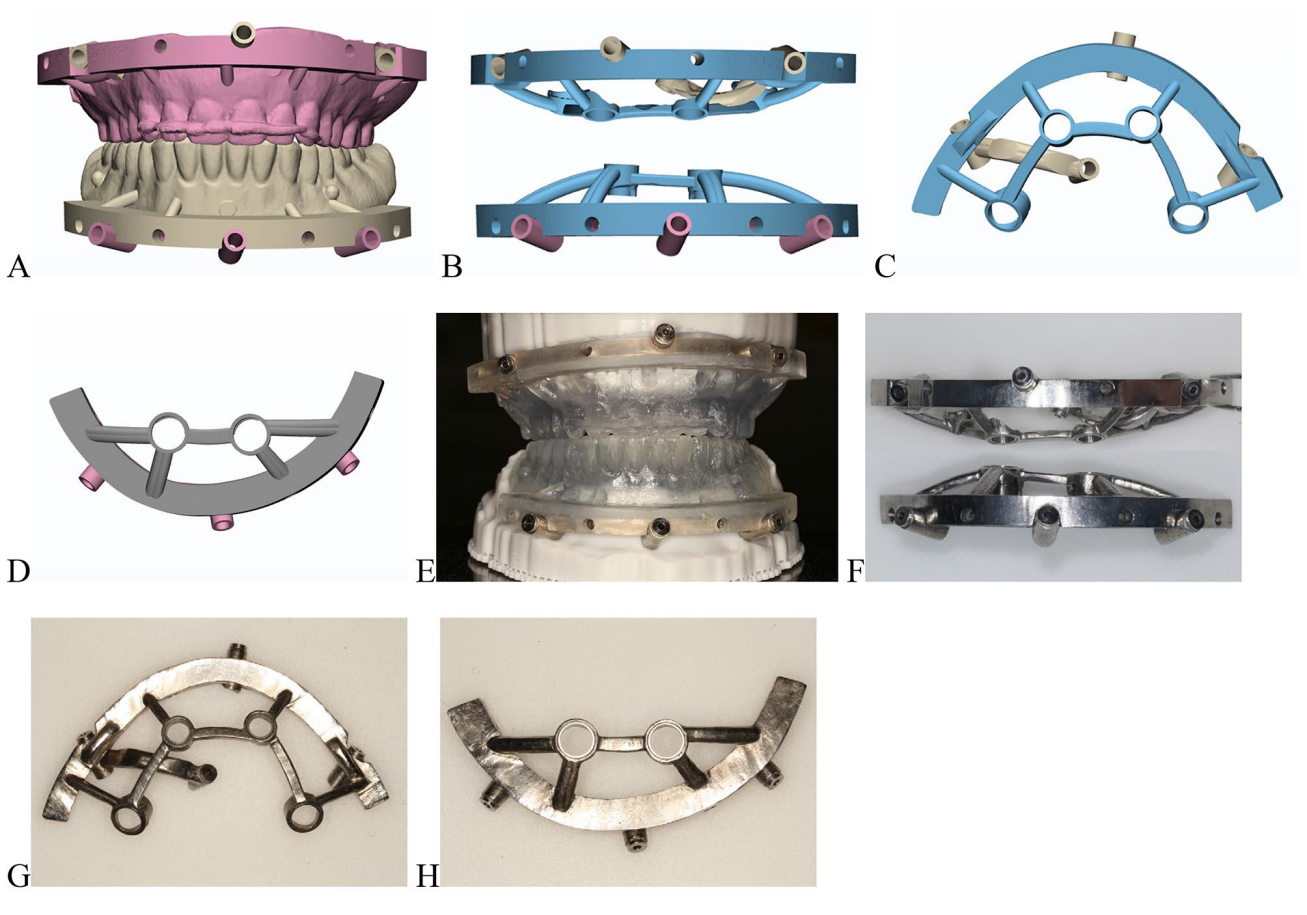
Digital designs and metal surgical templates. **A** and **E**, 3D-printed positioning temporary teeth. **B** and **F**, Cone-anchored metal stackable template in a horizontal direction. **C** and **G**, Maxillary template. **D** and **H**, Mandibular template
3. Manufacture the titanium alloy surgical templates with three-dimensional (3D) printing technology of selective laser melting (SLM) (Chamlion NCL-M2150T, China) (Fig. 2E-H). Design the cone-wedges and temporary teeth for assisted positioning (Materialise Magics 21.0), and 3D-print them using photosensitive resin (Heygears Surgical Guide UV resins, with Heygears Streamflow Dental 3D Printer) (Fig. 3).
  **Fig. 3:**
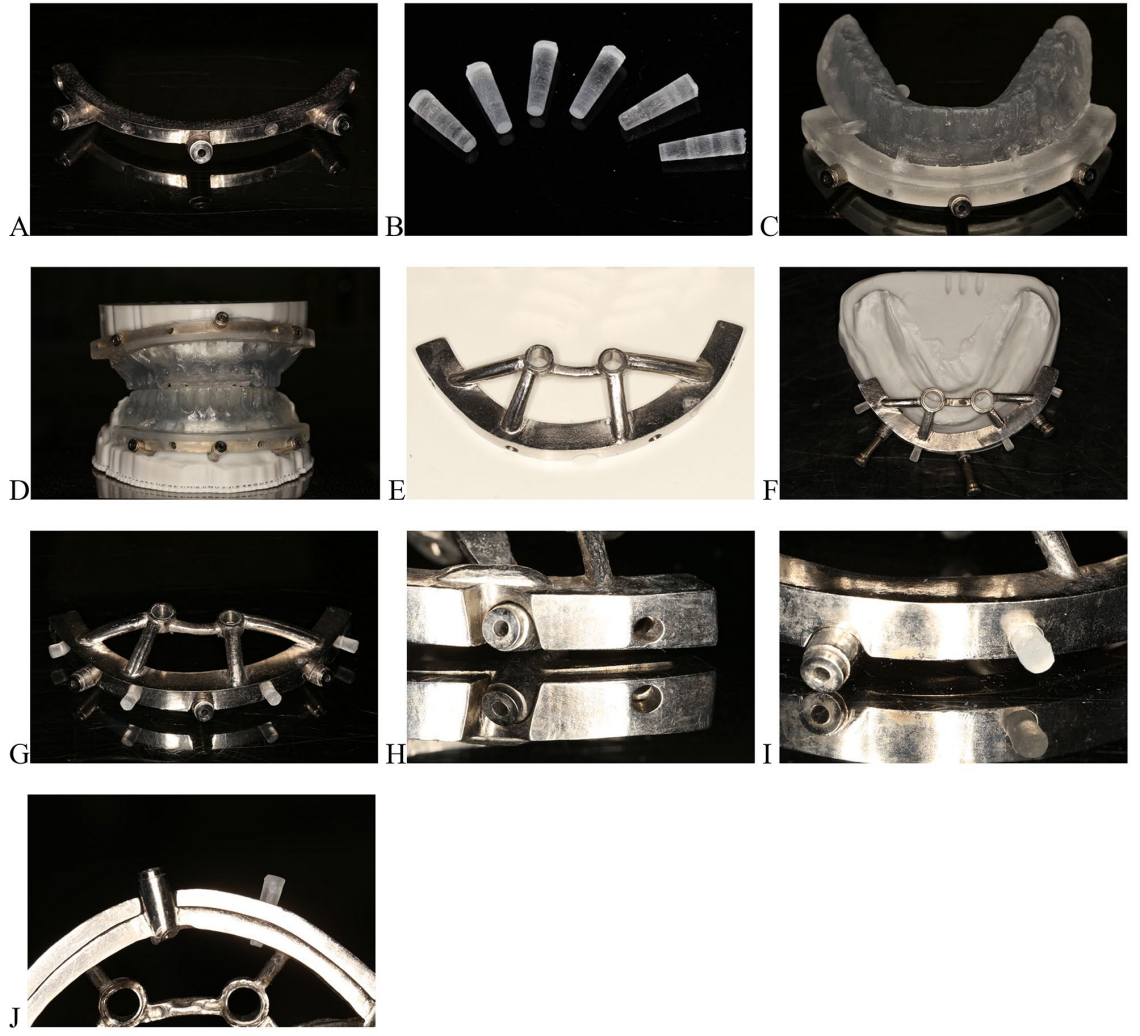
**A**, Mandibular base template. **B**, Cone-wedges. **C**, Mandibular 3D-printed positioning template. **D**, Assembled positioning template placed on the model (stackable guide 1). **E**, Mandibular implant placement template. **F**, Assembled implant placement template placed on the mandibular model (stackable guide 2). **G**, Assembled implant placement template. **H**, Insertion hole on the base template. **I**. **J**, **E**. **F**, Cones connecting the base template and implant placement templates in a horizontal direction (details of the assembled stackable guide)
4. Check the fit of the 3D-printed positioning templates and the base templates intraorally before surgery. Administer local anesthesia only at fixation pin sites. Use the positioning template and silicone bite to locate the metallic retainer base templates, and fit assembly pins into the maxillary and mandible (Fig. 4A, B). Administer local anesthesia at the implant sites after removing the positioning template. Make horizontal incisions at the crest of the alveolar ridge and vertical incisions at the middle of the arch, followed by flap elevation.
  **Fig. 4:**
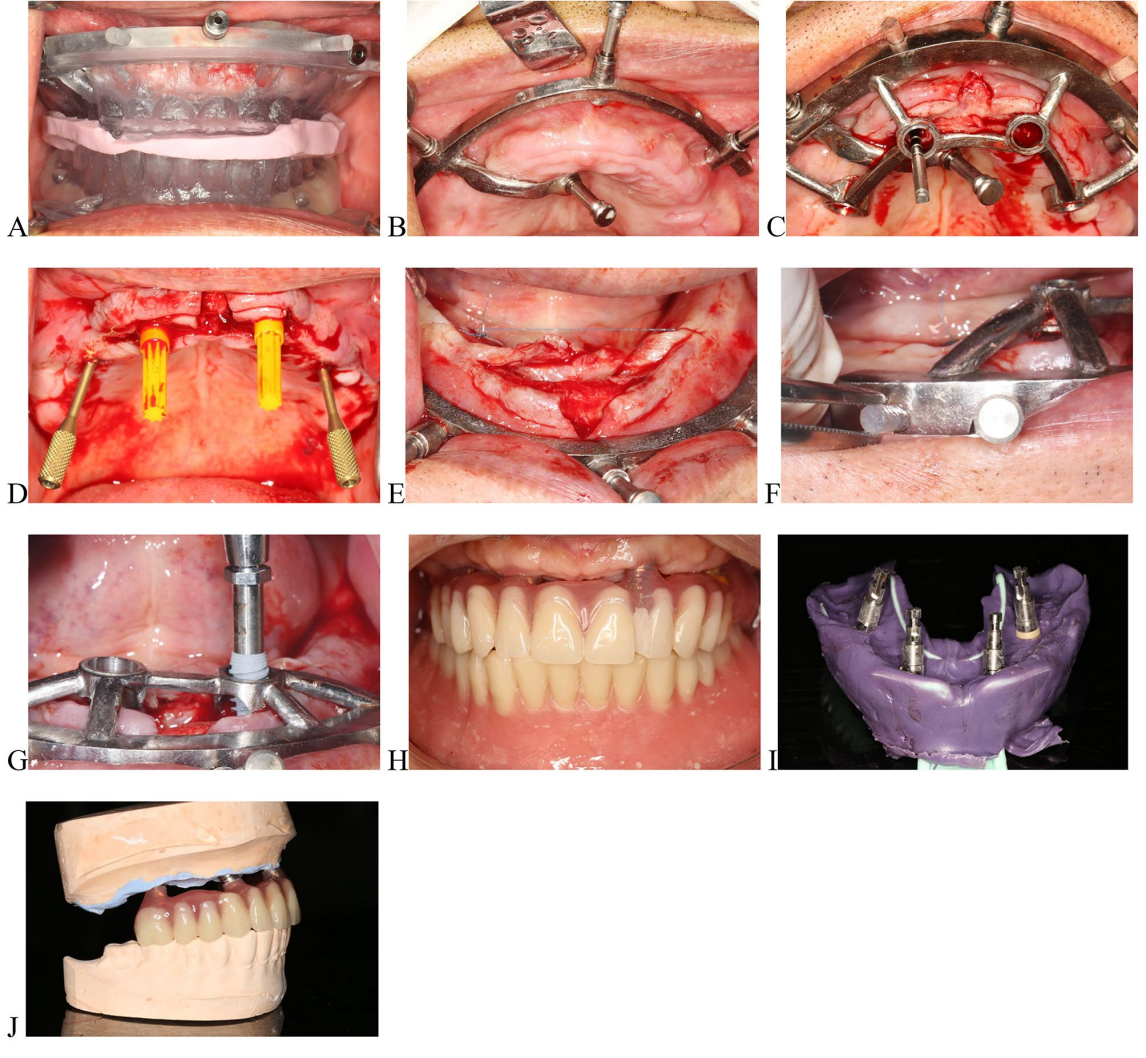
Intraoral photographs of the surgery. **A**, Positioning of 3D-printed positioning temporary teeth. **B**, Placement of maxillary retention template. **C**, Maxillary implant placement template was inserted into the base template by cone-wedges in a horizontal direction. **D**, Implant placement in maxillary. **E**, Placement of mandibular retention templates. **F**, Mandibular implant placement template was inserted into retention template by cones in a horizontal direction. **G**, Implant placement in mandible. **F**, Interim implant-supported complete prosthesis in maxillary and complete denture in mandible. **I**, The impression was taken with polyether materials. **J**, Interim implant-supported complete prosthesis in the maxillary model
5. Position and stabilize the metallic implant placement template on the base template using cones in a horizontal direction, and perform osteotomies following a preoperative-guided implant surgical plan (Fig. 4C). Place four dental implants (5 × 15 mm [2 in total], 4.3 × 13 mm [2 in total], Nobel Biocare Active) in the maxilla with an insertion torque of over 35 Ncm. Place the multi-unit abutments, close the wound, and take impressions with polyether materials (Fig. 4D, I).
6. Fabricate a new maxillary implant-supported fixed full-arch temporary prosthesis based on the pre-operative complete denture and maxillomandibular relations (Fig. 4H, J).
7. Position and stabilize the metallic implant placement template on the base template using four cone-wedges in the horizontal direction and make osteotomies following a preoperative-guided implant surgical plan (Fig. 4E, F). Place two dental implants (5 × 11.5 mm, Nobel Biocare Active) in the mandible under an insertion torque of > 35 Ncm (Fig. 4G). Finally, reline the mandible complete mandibular denture after removing the sutures.
8. Verify the occlusion and finish the temporary prosthesis.

## Discussion

Stackable surgical guides are popular among edentulous patients because they can avoid repeated removal of bone retainer pins [8, 13, 21–23]. However, retention and stability between the two elements of the stackable guide, which is separated into a base template and an upper-structure template, are crucial for surgical accuracy.

In previous reports, the anchoring mechanism was mainly classified as strut-inserted anchoring [13], snap anchoring [8, 17, 24], magnet anchoring [14–16], and pin-latch anchoring [18, 25]. Strut-inserted anchoring method requires high printing accuracy and the shrinkage characteristics of the printing material are also crucial. Snap anchoring method runs the risk of moving and rotating after being seated, like Locator® attachment system [26]. The magnet anchoring method leads to easy positioning attributed to the attraction force between magnets, but the strength of the attraction force affects the stability of the guides [14]. Pin-latch anchoring method can provide steady mechanical retention. The proposed technique uses a cone-wedge anchorage to connect the templates to the base template, which belongs to the pin-latch anchoring method. The cone-wedge design has several unique advantages. First, the cone-wedges were designed with a large diameter at one end and a small diameter at the other end (Fig. 3B) such that they could firmly clamp the base template and the upper-structure templates together by the force of friction. Second, it is more convenient to change the templates by inserting or removing cone-wedges of the same size into the retention holes. Third, the cone-wedges are easily positioned, even under the condition of minor misfits between the templates. However, there are fewer relevant studies on the connection types of stackable guides, and it is inconclusive which anchorage type is the most reliable.

Resin is the most commonly used material for a surgical guide, but it is easily deformed and requires a certain thickness to ensure strength [27]. The upgrade from resin to a metal 3D printing stackable guide provides better visibility of the alveolar ridge, operative space, and water cooling [13]. The proposed metal stackable guide includes three independent components: the base template which is fixed on the alveolar ridge by fixation pins; the occlusal positioning template (the upper structure 1); and the implant placement template (the upper structure 2). The upper structure templates were connected to the base template using several cone-wedges in a horizontal direction. Horizontally positioned wedges can strictly prevent the tilting of one end of the drilling template when the pressure from the drill and handpiece is applied vertically on the other end of the template. It was also found that this technique had better resistance to drill motion-caused loosening and better stabilization of the guide [28]. Therefore, this technique can accelerate operations and improve the patient experience.

The 6th International Team for Implantology Consensus Conference proceedings revealed a mean error of 1.2 mm at the entry point and 1.4 mm at the apical point, with a deviation of 3.5 degrees [25]. There was a significant difference in favor of partially edentulous cases compared with fully edentulous cases. The mean error for an entry point was 1.3 mm and that for the apex is 1.5 mm, with an angular deviation of 3.3 degrees, for fully edentulous cases [29]. Compared with other related studies, the additively manufactured, magnetically retained, and stackable resin implant surgical guide demonstrated implant angulation discrepancies ranging from 0 to 3.8 degrees, with 3D discrepancies at the coronal and apical region ranging from 0.07 to 1.73 mm and 0.07 to 1.91 mm, respectively [13]. The stackable restrictive surgical guides with a metal framework showed implant angulation discrepancies ranging from 3.19 to 9.99 degrees, and 3D discrepancies at the coronal and apical region ranged from 0.30 to 1.21 mm and 0.87 to 1.98 mm, respectively [15]. Here, the linear deviations in this technique ranged from 0.68 to 1.47 mm (mean = 0.97 mm) at the coronal implant region and from 0.78 to 2.14 mm (mean = 1.56 mm) at the apex. The angular deviations ranged from 0.98 to 4.62 degrees (mean = 2.95 degrees) (Table 1). According to a variation analysis between the virtual planning and the postplacement implant positions, the proposed technique produced fewer coronal implant deviations, and the apex and angular deviations were within the clinically acceptable range.

**Table 1:** The discrepancy between virtual and actual implant positions

Implant	Angle (Degree)	3D Coronal Implant Discrepancy(mm)	3D Apical Implant Discrepancy(mm)
16	3.01	0.68	1.45
13	0.98	0.79	0.78
24	1.53	1.47	1.70
26	3.28	1.08	1.74
34	4.62	1.21	2.14
44	4.29	0.60	1.53
Mean	2.95	0.97	1.56

However, the manufacture of a metal stackable surgical guide requires high precision and accuracy. And it is more expensive than resin materials, which may pose limitations for widespread clinical applications. Therefore, further research and development in SLM technology are required to overcome these issues.

## Summary

This article describes a novel cone-wedge anchored metal stackable surgical guide that aims to promote the templates’ stability and implant placement accuracy.

### Electronic supplementary material

Below is the link to the electronic supplementary material.

Supplementary Material 1

## Data Availability

The datasets used and/or analyzed during the current study are available from the corresponding author upon reasonable request.
