# Supplementary material for: Cone-wedge anchored surgical templates for stackable metal guide: a novel technique
Source: Int J Implant Dent. 2024 May 31;10:27. doi: 10.1186/s40729-024-00539-w (PMC11143131; doi:10.1186/s40729-024-00539-w)
Supplement: Supplementary file 1 — Supplementary Material 1 [file 40729_2024_539_MOESM1_ESM.docx]

**Reply letter**

February 16, 2024

Dear Editors,

We sincerely thank you and the reviewers for the valuable comments on our manuscript. Those comments are very helpful for us to revise and improve our paper. According to these comments, we have made extensive and careful revisions one by one and would like to resubmit it for your reconsideration. The revised portions are highlighted in red color in the revised paper. The details are shown in the following point-by-point.

Reviewer #1: first, please arrange as the results section cannot be found on this article, though the table is on.

Reply: Thank you for your valuable feedback. Because this article focused on a technical innovation rather than a clinical study or case report, the article structure was written in the form of a technical report. The results section was mainly arranged in the discussion section.

To better understand the advancements and effectiveness of our technique, we have incorporated the method and result sections into the abstract to provide a more comprehensive overview of our study.

“ABSTRACT

Objective: To address the instability in implant surgical guides, this technique proposes an alternative anchoring mechanism in the stackable metal surgical guides utilizing cone-wedge anchors for improved stability.

Methods: Postoperative implant position superimposed onto the preoperatively planned design using Mimics Medical 21.0 and Materialise Magics 24.0 to assess 3D coronal implant deviation, 3D apical implant deviation, and implant angular deviation.

Results: Postoperative cone-beam computed tomography (CBCT) revealed a high level of precision in the implant placement, with an average 0.97 mm deviation at implant coronal region, 1.56 mm at implant apexes, and 2.95° angular deviation.

Conclusion: This technique introduces a novel cone-wedge anchoring mechanism to enhance the stability of stackable metal surgical guide templates, addressing inherent instability issues. The utilization of this approach significantly improves the accuracy of implant placement procedures.”

Based on the information on the accuracy of this guide with the table, detail comparison with previous results on the reports should be mentioned.

Reply: We acknowledged the importance of providing a detailed comparison with previous reports on the accuracy of our guide. There are many articles reporting the accuracy of digital surgical guides, and we cited authoritative the 6th International Team for Implantology Consensus Conference proceedings results related to fully edentulous cases (“The mean error for an entry point was 1.3 mm and that for the apex is 1.5 mm, with an angular deviation of 3.3 degrees, for fully edentulous cases. **Ref 25**”). Besides, we added studies about stackable implant surgical guide deviation in the **Discussion part**.

“Compared with other related studies, the additively manufactured, magnetically retained, and stackable resin implant surgical guide demonstrated implant angulation discrepancies ranging from 0 to 3.8 degrees, with 3D discrepancies at the coronal and apical parts ranging from 0.07 to 1.73 mm and 0.07 to 1.91 mm, respectively [13]. The stackable restrictive surgical guides with a metal framework showed implant angulation discrepancies ranging from 3.19 to 9.99 degrees, and 3D discrepancies at the coronal and apical parts ranged from 0.30 to 1.21 mm and 0.87 to 1.98 mm, respectively [15].”

Also the cost of this should be politely mentioned, not only 'expensive' in this article.
Reply: The cost of a stackable metal guide is more or less 2 times more expensive than a regular implant surgical guide in China. However, the exact price varies from country to country due to market pricing and price differences.

Reviewer #2: The paper, "Cone-wedge anchored surgical templates for stackable metal guide: A novel technique," is interesting in that it describes how the layering of two separate templates, the "base template" and the "upper structure". They describe it is possible to perform full-mouth immediate loading implants with greater precision. However, the texts and figures are not clear in many respects and need to be revised and added. what the author's trying to say is generally clear, but the details are not, and even if the reader reads the "techniques" part, no one can reproduce the method. The specific points for modification and supplementation are outlined as follows:

Thanks so much for your positive comments on our work. We have carefully revised our manuscript following your advice and answered the questions point by point as follows. And we also have made corresponding modifications in the main text or supporting information carefully.

**Introduction**

Line 3: Isn't "and" after [4] grammatically unnecessary?

Reply: We are very sorry for our careless mistake. We have promptly rectified this error by removing the unnecessary conjunction.

Line 13: "For full-arch edentulous..." It is difficult to understand the sentence. What does "to fix the alveolar bone using retainer pins" mean?

Reply: To make it clearer, we rephrased this sentence. Please see the revision in the **Introduction part**. We hope this modification addressed your concerns.

“Sequential implant surgical templates are typically utilized for full-arch edentulous patients, which are made up of several subset templates to function in scenarios such as template position, bone reduction, or implant placement. These subset templates share the same retainer pins anchored on the alveolar bone.”

**Technique**

Throughout, surgical techniques such as infiltration anaesthesia, incision, dissection and suturing are not described.N

Reply: We appreciate your suggestion, and we have now added detailed descriptions of these surgical techniques to enhance the comprehensiveness of the content (**Technique No.4 and No.5**).

“4. Check the fit of the 3D-printed positioning templates and the base templates intraorally before surgery. Administer local anesthesia only at fixation pin sites. Use the positioning template and silicone bite to locate the metallic retainer base templates, and fit assembly pins into the maxillary and mandible (Figure 4A, B). Administer local anesthesia at the implant sites. Make horizontal incisions at the crest of the alveolar ridge and vertical incisions at the middle of the arch, followed by flap elevation.

5. Position and stabilize the metallic implant placement template on the base template using cones in a horizontal direction, and perform osteotomies following a preoperative-guided implant surgical plan (Figure 4C). Place four dental implants (5×15mm [2 in total], 4.3×13mm [2 in total], Nobel Biocare Active) in the maxilla with an insertion torque of over 35 Ncm. Place the multi-unit abutments, close the wound, and take impressions with polyether materials (Figure 4D).”

No.1

Line 10-13:"a diagnosis deture" is NOT showm in figure 1 A～C. The content of the text differs from that of the figure 1.

Reply: We apologize for the confusion. Thank you for bringing to our attention the discrepancy between the text and Figure 1 (A-C) regarding the absence of "a diagnosis denture" in the figures. To address this issue, we have added photographs of the diagnostic denture in the revised manuscript.

Figure 1D. Maxillary diagnosis denture with smile line.

Figure 1E. Mandibular diagnosis denture

No.2

Figure2: Why are the holes for implant placement in D and H different sizes?

Reply: This problem comes from two main reasons. The first is that the Figure 2D mandibular template digital design is not the final version, and we have replaced it with a new one. The second is that Figure 2H was taken from the tissue side view, and we have also replaced it. Please see the revised version.

Figure 2D. Mandibular template design file

Figure 2H. Mandibular template

F is as in not horizontal direction.

Reply: We have addressed your concern regarding the orientation of image F. We have retaken the photograph and uploaded the revised image accordingly.

Figure 2F. Cone-anchored metal stackable template in a horizontal direction

Isn't G opposite left to right compared to C?

Reply: Thank you for your insightful observation. We've noted the discrepancy in the left-to-right orientation of images G and C and have corrected it for consistency. The updated images have been uploaded.

Figure 2G. Maxillary template

No.3

The "cone wedge" is probably the most important part of the paper, but it is a shame that Figure 3 is so difficult to understand. Can you show it more clearly? What does the red arrow indicate?

Reply: We have renewed Figure 3 to improve its clarity (See the revised manuscript). Additionally, we have added caption explanations to elucidate the fixation method of the stackable guide. We hope this clarification and modification address your concerns. If you require further explanation or have any additional questions, please feel free to let us know.

A B C

D E F

G H I J

Fig 3.A, Mandibular base template. B, Cone-wedges. C, Mandibular 3D-printed positioning template. D, Assembled positioning template placed on the model (stackable guide 1). E, Mandibular implant placement template. F, Assembled implant placement template placed on the mandibular model (stackable guide 2). G, Assembled implant placement template. H, Insertion hole on the base template. I. J, E. F, Cones connecting the base template and implant placement templates in a horizontal direction (details of the assembled stackable guide).

Base template

Postioning template

Cone-wedges

Assembled template 1

Stackable guide 1 is composed of the base template, positioning template, and cone-wedges, acting to seat the base template on the alveolar bone.

Base template

Implant placement template

Cone-wedges

Assembled template 2

Stackable guide 2 is composed of the seated base template, implant placement templates, and cone-wedges, acting to place the implants precisely.

The templates have several holes that enable the cone-wedges to be inserted horizontally to stabilize the stackable guides.

No.4

"The words 'positioning template', 'retainer template', 'implant placement template' and 'base template' suddenly appear and are very difficult to understand." Wouldn't it be easier for readers to understand if the three components in the part of "discussion" were explained here?
Reply: Thank you for your suggestion. To make it easier for readers to understand the structure of a stackable surgical template, we have revised and added a detailed explanation in the **Introduction part**.

"Typically, a stackable surgical template can be divided into two parts: the base template and the super-structure templates. The base template is connected to the alveolar bone by retainer pins, and the super-structural templates need to connect to the base template. According to its function in surgery, the super-structural templates can be categorized into several subset templates such as positioning template, bone reduction template, and implant template. A stackable surgical template takes the strategy that that the base template is precisely fixed on the alveolar bone by bone retainer pins with the help of the positioning template, and the upper-structure templates (such as the positioning template, the bone reduction template, and implant placement template) are anchored to the base template, correspondingly."

Why can't we see the holes for the placement of the two distal implants in Figure 4C?
Reply: Thank you for bringing to our attention the absence of holes for the placement of the two distal implants in Figure 4C. We apologize for the confusion this may have caused. We have replaced the incorrect picture with the correct surgical guide process picture as follows.

Figure 4C. Maxillary implant placement template was inserted into the base template by cone-wedges in a horizontal direction.

Figure 4D does not show impression taking.

Reply: We appreciate your attention to detail. Figure 4D was taken after the four implants were placed, and it can demonstrate implantation direction. The impression was taken with polyether materials, and we have uploaded the image (Figure 4I).

Figure 4I. The impression was taken with polyether materials.

The figure legend for H is misprinted as "F".

Reply: We sincerely apologize for the mistake in the figure legend for image H. The correction has been promptly made, and the figure legend now accurately corresponds to image H.

No.5

It does not state how the temporary prosthesis was made.

Reply: Thank you for your feedback regarding the absence of information on how the temporary prosthesis was fabricated. We emphasize that the focus of our article is on the accuracy of the metal stacking guide. Therefore, we did not extensively discuss the process of fabricating the temporary prosthesis. The temporary prosthesis was made by traditional methods: an impression was taken, and a technician fabricated it using the original diagnostic denture. We added a temporary prosthesis picture in Figure 4J.

Figure 4J. Interim implant-supported complete prosthesis in the maxillary model.

No.6

When was the base template of the mandible fitted?

Reply: Thank you for your question. At the beginning of the surgery, we fitted the base template of the mandible using a bite registration guide and secured it with fixation pins. This process was carried out concurrently with the fixation of the base templates for both the upper and lower jaws.

Is the prosthesis producted immidiately after surgery convetional complete denture or implant overdenture? How has your new technique helped you to produce it?

Reply:

In the maxilla, an immediate temporary prosthesis was fabricated using traditional edentulous maxillary impressions and the original diagnostic denture, which was remolded chairside by a technician.

In the mandible, we chose submerged healing because we only placed two implants as required by the patient. As a result, the immediate post-operative prosthesis remains the

a conventional complete denture in the mandible.

Our new technique focuses on enhancing the strength of the surgical guide, reducing the size of the guides for a better surgical field of vision, optimizing the ease of implant surgery, and improving the precision of implant placement. However, it did not directly assist in the production of the immediate prosthesis.

**Discussion**

Page 1

Line1: At least I do not think stackable surgical guides are POPULAR for edentulous patients. Are there any studies to support your statement?

Reply: Thank you for your question regarding the popularity of stackable surgical guides for edentulous patients.

As mentioned in our manuscript, the advantages of stackable guides have been increasingly recognized in recent years. We have supplemented our discussion with relevant literature [8,13,21,22,23] supporting this trend.

Line14:As mentioned above, the "cone-wedge design" is an important point for your paper, but the problem is that it is difficult for readers to understand how it works.Figure 3 needs to be made easier to understand.

Reply: Thank you for your valuable comment. We have renewed Figure 3 to improve its clarity (Please see the revised manuscript). Additionally, we have added caption explanations to elucidate how the “cone-wedge design” works. Please see the following schematic figures.

Base template

Postioning template

Cone-wedges

Assembled template 1

Stackable guide 1 is composed of the base template, positioning template, and cone-wedges, acting to seat the base template on the alveolar bone.

Base template

Implant placement template

Cone-wedges

Assembled template 2

Stackable guide 2 is composed of the seated base template, implant placement templates, and cone-wedges, acting to place the implants precisely.

The templates have several holes that enable the cone-wedges to be inserted horizontally to stabilize the stackable guides.

Page 2

Line6: Wouldn't it be easier for the reader to understand the description of the components of your proposed stackable guide if they were placed in the 'Techniques' section?

Reply: Thank you for your valuable suggestion. We have provided a detailed introduction to the stackable guide in the introduction section. Additionally, we have made comprehensive modifications to Figure 3 and added detailed annotations. Therefore, we believe that repeating this information in the techniques section is unnecessary. We sincerely appreciate your feedback.

"Typically, a stackable surgical template can be divided into two parts: the base template and the super-structure templates. The base template is connected to the alveolar bone by retainer pins, and the super-structural templates need to connect to the base template. According to its function in surgery, the super-structural templates can be categorized into several subset templates such as positioning template, bone reduction template, and implant template. A stackable surgical template takes the strategy that the base template is precisely fixed on the alveolar bone by bone retainer pins with the help of the positioning template, and the upper-structure templates (such as the positioning template, the bone reduction template, and implant placement template) are anchored to the base template, correspondingly."

Line9:"The upper structures can..." sentence should be altered, together with Figure 3, to make it easier to understand.

Reply: Thank you for your valuable suggestion. We have revised both the sentence in line 9 and Figure 3 to ensure consistency and enhance clarity, making it easier for readers to understand. Any confusion was welcomed to feedback to us.

“The upper structure templates were connected to the base template using several cone-wedges in a horizontal direction.”

A B C

D E F

G H I J

Fig 3.A, Mandibular base template. B, Cone-wedges. C, Mandibular 3D-printed positioning template. D, Assembled positioning template placed on the model (stackable guide 1). E, Mandibular implant placement template. F, Assembled implant placement template placed on the mandibular model (stackable guide 2). G, Assembled implant placement template. H, Insertion hole on the base template. I. J, E. F, Cones connecting the base template and implant placement templates in a horizontal direction (details of the assembled stackable guide).

Line12: What evidence is there to state that this technique is superior in its resistance to resonance-induced loosening?

Reply: Thank you for your inquiry regarding the evidence supporting the superior resistance of our technique to resonance-induced loosening.

In our response, we highlighted that while the guide sleeve effectively controls drilling motion in a completely limiting design, reported errors in guided surgeries stem from template movement during implant placement. We noted the precision demonstrated in both the usage and implantation phases of our guide. Additionally, we emphasized the effectiveness of horizontal cone wedges in stabilizing the guide template. To enhance the rigor and reader comprehension, we have supplemented our argument with relevant literature (**[28]. Hang-Nga M, Du-Hyeong L. Effects of supporting conditions and anchor microscrew on the stabilization of the implant guide template during the drilling process: An in vitro study. J Prosthet Dent 2020;124(6):727.e1-727.e8.**).

Consequently, we have modified the sentence to**:** "It was also found that this technique had better resistance to drill motion-caused loosening and better stabilization of the guide template."

Line20: "0.31" is different from the value in the table. It describes the difference between the preoperatively planned implant position and the actual position of the implants placed, but does not say how the measurements were taken at all. Please describe the method of measurement.
Reply: We sincerely apologize for the careless mistake. The linear deviations in this technique ranged from 0.68 to 1.47 mm (mean=0.97mm, SD=0.31mm) at the coronal implant region. Because we listed SD in the last row in the Excel table, we mistakenly wrote 0.31 as a linear deviation at the coronal implant region in the article. We have revised the corresponding correction in the manuscript.

The method of measurement is demonstrated as follows:

1. Split the preoperative CT with the radiographic guide into upper and lower jaws, and fit it to the postoperative CT model using the bony landmarks.

2. Referring to the double scanning process, the guide was fitted to the preoperative CT model, and at this time, the guide plate was simultaneously positioned in the postoperative CT model.

3. Separate the real position of the implant on the postoperative CT model and reconstruct the preoperative design position according to the guide plate.

4. Trim the model, measure the points, and calculate the relevant indexes according to the coordinates. (Software: Mimics Medical 21.0, Materialise Magics 24.0)

Yours sincerely,

Yi Zhou

The State Key Laboratory Breeding Base of Basic Science of Stomatology (Hubei-MOST) & Key Laboratory of Oral Biomedicine Ministry of Education, Department of Dental Implantology, School & Hospital of Stomatology, Wuhan University, Wuhan, China

E-mail: dryizhou@whu.edu.cn
